# The efficacy and safety of auricular point combined with moxibustion for insomnia: A protocol for systematic review and meta-analysis

**DOI:** 10.1097/MD.0000000000022107

**Published:** 2020-10-09

**Authors:** Rui Jin, Xu Wang, Yubing Lv, Guangnan Xu, Chen Yang, Yang Guo, Xinju Li

**Affiliations:** a Tianjin University of Traditional Chinese Medicine; b Tianjin Academy of Traditional Chinese Medicine Affiliated Hospital; c National Clinical Research Center for Chinese Medicine Acupuncture and Moxibustion; d First Teaching Hospital of Tianjin University of Traditional Chinese Medicine, Tianjin, China.

**Keywords:** auricular point, insomnia, moxibustion, protocol, systematic review

## Abstract

**Background:**

Insomnia is a common sleep disorder, which seriously affects people's quality of life and work ability. In China, auricular therapy and moxibustion therapy have a long history in treating insomnia. Clinical studies have shown that auricular point and moxibustion can effectively improve insomnia symptoms. At present, auricular point combined with moxibustion in the treatment of insomnia has been widely used in China, but its overall effectiveness and safety are still unclear. There is a lack of systematic evaluation of auricular point combined with moxibustion in the treatment of insomnia. This paper aims to evaluate the efficacy and safety of auricular point combined with moxibustion in the treatment of insomnia.

**Methods:**

Retrieve randomized controlled trials of auricular point combined with moxibustion from PubMed, EMbase, Cochrane Library, Web of Science, China National Knowledge Infrastructure, WanFang, the Chongqing VIP Chinese Science and Technology Periodical Database, and China biomedical literature database from their establishment to August 2020. Search Baidu Scholar, Google Scholar, International Clinical Trials Registry Platform, and Chinese Clinical Trials Registry for unpublished gray literature. Two researchers independently applied RevMan 5.3 software for data extraction and risk assessment of bias.

**Results:**

This study evaluated the efficacy and safety of auricular point combined with moxibustion in the treatment of insomnia from Pittsburgh sleep quality index, Rhone planck sleepiness scale, Traditional Chinese medicine syndrome scores, Hamilton anxiety scale (HAMA), Hamilton Depression, 5-hydroxytryptamine, incidence of adverse reactions, and other aspects.

**Conclusion:**

This study will provide theoretical support for the clinical application of auricular point combined with moxibustion in the treatment of insomnia.

**Ethics and dissemination:**

The private information from individuals will not publish. This systematic review also will not involve endangering participant rights. Ethical approval is not required. The results may be published in a peer-reviewed journal or disseminated in relevant conferences.

**OSF Registration number:**

DOI 10.17605/OSF.IO/8VZRJ.

## 1 Introduction

Insomnia is a common disease of difficulty in falling asleep or sleep disorder, which leads to decreased sleep time or decreased sleep quality. With the acceleration of social rhythm and the aggravation of life pressure, the incidence of this disease is 15% to 25%,^[1]^ and it tends to be young.^[2]^ It is generally believed that environmental factors, physical factors and psychological factors are closely related.^[3]^ Long-term insomnia will seriously affect the quality of life or social activities of patients, and may also induce other chronic organic lesions^[4]^ and mental disorders,^[5]^ causing heavy economic burden on families and society. At present, western medicine mostly uses oral sedative-hypnotics to treat insomnia, but long-term use of western medicine has certain dependence and adverse reactions.^[6]^ Auricular therapy and moxibustion, as the traditional Chinese medicine characteristic external therapy, have the characteristics of safety, less side effects, and good acceptability. Current clinical studies have shown that auricular acupuncture treatment for insomnia can promote the blood flow speed of bilateral vertebral artery and basilar artery of the brain and increase the blood flow supply of the brain, so as to relieve insomnia symptoms such as dizziness and dreaminess.^[7]^ Moxibustion plays the role of warming and dredging the meridians, harmonizing Yin and Yang, and significantly improving the quality and efficiency of sleep.^[8]^ Auricular point combined with moxibustion is effective in the treatment of insomnia, but there are differences between the design and efficacy of existing clinical trials, and lack of systematic evaluation. This study plans to systematically evaluate the efficacy and safety of auricular point combined with moxibustion in the treatment of insomnia.

## 2 Methods

### 2.1 Protocol register

This protocol of systematic review and meta-analysis has been drafted under the guidance of the preferred reporting items for systematic reviews and meta-analyses protocols (PRISMA-P). Moreover, it has been registered on open science framework (OSF) on August 8, 2020 (Registration number: DOI 10.17605/OSF.IO/8VZRJ).

### 2.2 Ethics

Ethical approval is not required because there is no patient recruitment and personal information collection, and the data included in our study are derived from published literature.

### 2.3 Inclusion criteria

#### 2.3.1 Type of studies

It included randomized controlled trials of auricular point combined with moxibustion for insomnia and languages are English and Chinese only.

#### 2.3.2 Research objects

All included cases meet the diagnostic criteria for insomnia,^[9]^ regardless of nationality, race, age, gender, course of disease, etc.

#### 2.3.3 Intervention types

The control group was treated with conventional western medicine, including benzodiazepines (such as Diazepam) and non-benzodiazepines (such as Zolpidem), with no limit on the types and doses. The treatment group uses auricular point combined with moxibustion, the points of auricular therapy and moxibustion, the operation methods, as well as the treatment frequency, treatment time and so on are not limited.

#### 2.3.4 Observation indexes

1. Primary outcome: Pittsburgh sleep quality index.
2. Secondary outcomes: Rhone Planck sleepiness scale; Traditional Chinese medicine syndrome scores; Hamilton anxiety scale (HAMA); Hamilton depression; 5-hydroxytryptamine (5-HT); Incidence of adverse reactions.

### 2.4 Exclusion criteria

1. Literatures that were abstracts, had incomplete data, or whose required data could not be obtained after contacting the author;
2. For repeated literatures, choosing the one with the most complete information;
3. Literatures with obvious errors in the data;
4. Literatures with the treatment group applied auricular point combined with moxibustion as well as using other treatment methods, such as western medicine, acupuncture, traditional Chinese medicine, etc.

### 2.5 Search strategy

Randomized controlled trials of auricular point combined with moxibustion for insomnia were searched in PubMed, EMbase, Cochrane Library, Web of Science, China National Knowledge Infrastructure, WanFang, the Chongqing VIP Chinese Science and Technology Periodical Database, and China biomedical literature database. The retrieval time was from their establishment to August 2020. At the same time, search Baidu, Google Scholar, International Clinical Trials Registry Platform, and Chinese Clinical Trials Registry for unpublished gray literature. Chinese retrieval terms were: auricular point, auricular acupuncture, moxibustion, insomnia, etc. English retrieval terms were moxibustion, Ear Acupuncture, insomnia, etc. The search strategy (PubMed) is shown in Table 1.

**Table 1:**
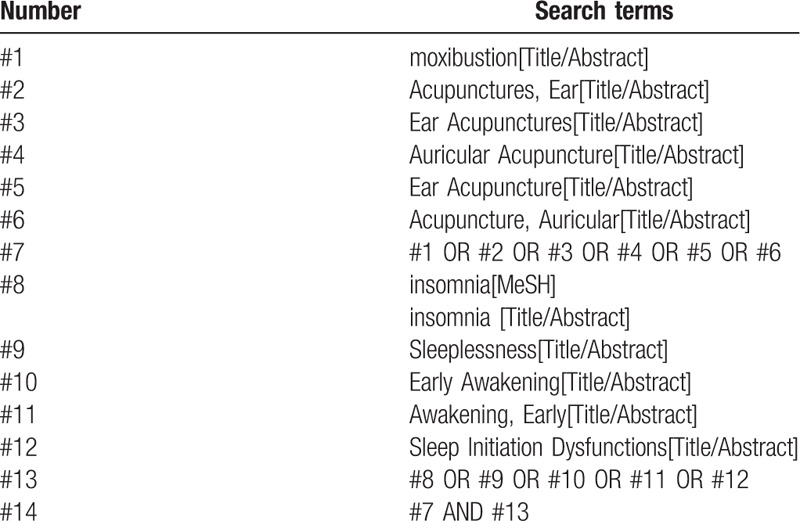
Search strategy in PubMed database.

### 2.6 Data extraction principles

**Figure 1:**
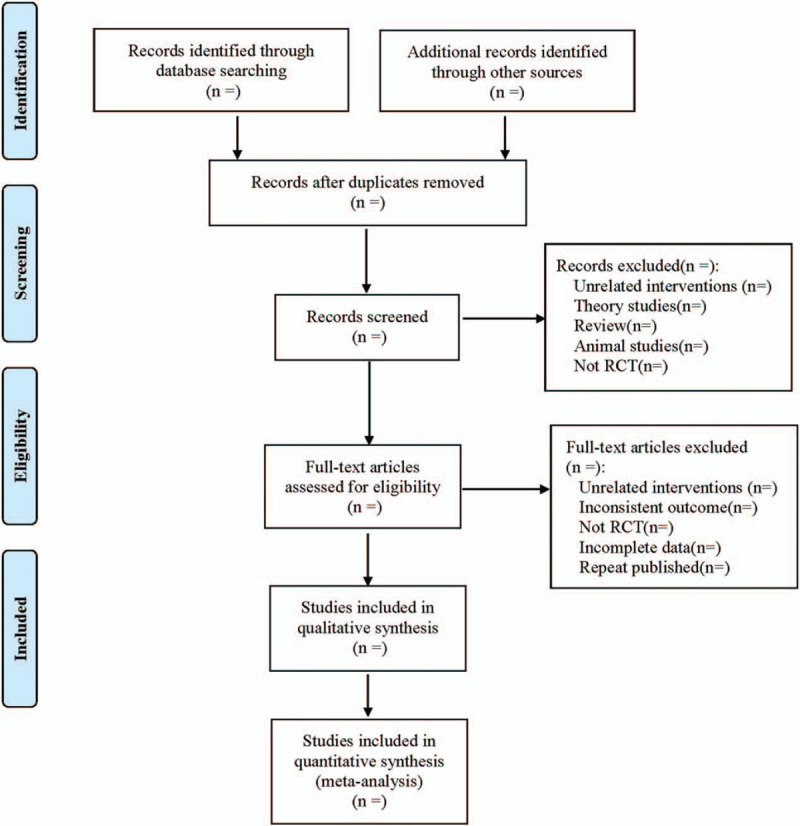
Flow diagram.

The Endnote X7 literature management software was used by the 2 researchers to independently screen, extract, and include literature data according to literature quality evaluation criteria of the Cochrane Collaboration Handbook (Version 5.0), and the reasons for the exclusion were recorded. If there were conflicts about articles, we would consult with a third researcher for assessments. Excel 2019 literature information database was established to extract data. The following information will be extracted:

1. Basic information of articles: title, first author, year of publication, sample size, sex ratio, average age, average course of disease;
2. Intervention measures: name, dose, and course of treatment of western medicine in the control group, the acupoints of moxibustion and auricular therapy, treatment frequency and course of treatment in the treatment group.
3. The entry of risk of bias assessment.
4. The outcomes and relevant measurement data. The literature screening process is shown in Figure 1.

### 2.7 Literature quality evaluation

The risk of bias for each eligible study will be assessed by 2 researchers respectively according to the Cochrane Collaboration's tool including 7 terms. According to these criteria (random sequence generation, allocation concealment, blinding, incomplete data, selective result reports, and other bias), risk of bias is classified into the following levels: unclear, low, and high risk of bias. Any divergences will be solved through discussion by a third researcher.

### 2.8 Statistical analysis

#### 2.8.1 Data analysis and processing

The RevMan 5.3 software provided by the Cochrane Collaboration was used for statistical analysis. For dichotomous variables, relative risk was used for statistics. For continuous variables, weighted mean difference was selected when the tools and units of measurement indicators are the same, Standardized mean difference was selected with different tools or units of measurement, and all the above were represented by effect value and 95% confidence interval (CI). Heterogeneity test: Q test was used to qualitatively determine inter-study heterogeneity. If *P* ≥ .1, there was no inter-study heterogeneity, If *P* < .1, it indicated inter-study heterogeneity. At the same time, *I*^*2*^ value was used to quantitatively evaluate the inter-study heterogeneity. If *I*^*2*^ ≤ 50%, the heterogeneity was considered to be good, and the fixed-effect model was adopted. If *I*^*2*^ > 50%, it was considered to have significant heterogeneity, the source of heterogeneity would be explored through subgroup analysis or sensitivity analysis. If there was no obvious clinical or methodological heterogeneity, it would be considered as statistical heterogeneity, and the random-effect model would be used for analysis. Descriptive analysis was used if there was significant clinical heterogeneity between the 2 groups and subgroup analysis was not available.

#### 2.8.2 Dealing with missing data

If data is missing or incomplete, we will contact the corresponding author to obtain the missing data. If not, this study will be removed.

#### 2.8.3 Heterogeneity and subgroup analysis

In order to eliminate the clinical heterogeneity between studies, subgroup analyses were conducted according to the types of western medicine and the course of the disease. At the same time, according to the classification of auricular point treatment, it could be divided into 2 subgroups: sticking pills on auricular points and auricular acupuncture treatment.

#### 2.8.4 Sensitivity analysis

In order to test the stability of meta-analysis results of outcomes, a one-by-one elimination method will be adopted for sensitivity analysis.

#### 2.8.5 Reporting bias

For the major outcome indicators, if the included study was ≥10, funnel plot was used to qualitatively detect publication bias. Egger and Begg tests are used to quantitatively assess potential publication bias.

#### 2.8.6 Evidence quality evaluation

The Grading of Recommendations Assessment, Development, and Evaluation will be used to assess the quality of evidence. It contains 5 domains (bias risk, consistency, directness, precision, and publication bias). And the quality of evidence will be rated as high, moderate, low, and very low.

## 3 Discussion

Insomnia belongs to the categories of “sleepless (Bu Mei)” and “can’t sleep (Bu De Mian)” in Traditional Chinese medicine. Its pathogenesis is the disharmony between Ying and Wei as well as the imbalance between Yin and Yang. People cannot sleep because of the disharmony between qi and blood in the body, while Yin and Yang is not intersecting, causing the failure of Yang entering into Yin. Insomnia is closely related to the heart, liver, spleen, and kidney. The main syndromes are deficiency of both heart and spleen, liver depression transforming into fire and hyperactivity of fire due to Yin deficiency, so the treatment should be to balance the Yin and Yang of zang-fu viscera.

According to Traditional Chinese medicine, the ear is the most densely distributed part of the body's channels and collaterals, and it is closely related to the zang-fu viscera and collaterals. By stimulating the auricular points, the channels and collaterals can be dredged, qi and blood can be regulated, so as to achieve the purpose of regulating the balance of Yin and Yang of the zang-fu viscera and treating diseases. Auricular acupoints commonly used to treat insomnia are: CO_15_ (Xin), TF_4_ (Shenmen), AT_4_ (Pizhi Xia), AH_6a_ (Jiaogan), etc. CO_15_ (Xin) has the effect of nourishing blood and the heart and calming the heart and the mind; TF_4_(Shenmen) has the effect of calming the mind and invigorating qi and strengthening the heart.^[10]^ AT_4_(Pizhi Xia) can calm the mind and harmonize Yin and Yang; AH_6a_(Jiaogan) can coordinate cerebral cortex excitatory and inhibitory dysfunctions. Studies have shown that auricular therapy can enhance parasympathetic excitability and inhibit sympathetic abnormal excitability.^[11]^ Moxibustion stimulates the meridians and acupoints through the burning of moxa, which can dredge the meridians, stimulate the functions of zang-fu viscera, warm the meridians, relieve cold and pain, harmonize Yin and Yang, and strengthen the body. Points commonly used to treat insomnia by moxibustion are: GV20(Baihui), KI1(Yongquan), KI3(Taixi), etc. GV20(Baihui) belongs to Governor vessel and is located on the top of the head. It can nourish and calm the mind and nourish qi and strengthen the brain. KI1(Yongquan)is located in the sole of the foot, which has the effect of restoring normal coordination between heart and kidney and conducting fire back to its origin.^[12]^ KI3 (Taixi) is the source of nourishing the viscera of the human body and has the functions of nourishing the Yin and benefiting the kidney and nourishing the heart and calming the mind.^[13]^ Experimental studies confirmed that moxibustion can increase 5-HT and 5-hydroxyindoleacetic acid in the hypothalamus of rats, lower dopamine (DA) and norepinephrine levels, and regulate sleep state of rats.^[14]^

Previous studies showed that the effective rate of oral sedative-hypnotics was 70.83% to 73.33%. The effective rate of auricular point combined with moxibustion treating insomnia is 89.58% to 93.33%,^[15,16]^ which is significantly higher than that of western medicine alone. The curative effect of auricular acupuncture point combined with moxibustion is long-lasting and safe, and there will be no psychological dependence, withdrawal symptoms, relapse,^[17]^ and so on. Therefore, it is necessary to evaluate the evidence of auricular point combined with moxibustion in the treatment of insomnia, objectively evaluate the clinical efficacy and safety of auricular point combined with moxibustion, and provide feasible and effective therapeutic means for clinical practice.

Meanwhile, this study has some limitations: the included literatures are only in Chinese and English, and there may be some publication bias. In many studies, the implementation process of allocation concealment is not clear and has certain bias. In addition, there may be some clinical heterogeneity due to the different acupoints used in auricular therapy and moxibustion.

## Author contributions

**Data collection:** Yubing Lv and Guangnan Xu.

**Funding to support:** Yang Guo and Xinju Li.

**Literature search:** Rui Jin and Wang Xu.

**Software operation:** Chen Yang.

**Supervision:** Yang Guo and Xinju Li.

**Writing – original draft:** Rui Jin and Wang Xu.

**Writing – review & editing:** Rui Jin, Wang Xu and Yang Guo.

## Abbreviations

Abbreviations: 5-HT = 5-hydroxytryptamine, CI = confidence interval, DA = dopamine, HAM = Hamilton anxiety scale A, OSF = open science framework, PRISMA-P = preferred reporting items for systematic reviews and meta-analyses protocols.
